# Real-World Technology Use Among People With Mental Illnesses: Qualitative Study

**DOI:** 10.2196/10652

**Published:** 2018-11-23

**Authors:** Elizabeth Carpenter-Song, Valerie A Noel, Stephanie C Acquilano, Robert E Drake

**Affiliations:** 1 Department of Anthropology Dartmouth College Hanover, NH United States; 2 Westat Lebanon, NH United States; 3 The Dartmouth Institute for Health Policy and Clinical Practice Geisel School of Medicine at Dartmouth Lebanon, NH United States

**Keywords:** qualitative research, technology, mental health, mobile phone

## Abstract

**Background:**

There is growing interest in using technology-based tools to support mental health recovery. Yet, despite evidence suggesting widespread access to technology among people with mental illnesses, interest in using technology to support mental health, and effectiveness of technology-based tools developed by researchers, such tools have not been widely adopted within mental health settings. Little is currently known about how mental health consumers are using technology to address mental health needs in real-world settings outside of controlled research studies.

**Objective:**

This qualitative study examined current practices and orientations toward technology among consumers in 3 mental health settings in the United States.

**Methods:**

Ethnographic observations and semistructured interviews were conducted. Observations focused on if and how technology was salient within the setting and documented relevant behaviors, interactions, and dialogue in fieldnotes. Ethnographic data informed the development of a semistructured interview that inquired into technology use and interest among consumers (n=15) in a community mental health setting. Fieldnotes and interview transcripts were reviewed and coded by multiple researchers. Key concepts and patterns identified were refined by the research team to develop the main findings.

**Results:**

Ownership of technology, although common, was not ubiquitous and was varied across the sites. Participants had varying levels of awareness regarding the key capabilities of modern technologies. Participants used technology for many purposes, but there was limited evidence of technology use to support mental health. Technology-based tools specific to mental health were not routinely used, although some participants found widely available mobile apps to be helpful in recovery.

**Conclusions:**

Qualitative findings suggest that many, but not all, clients will be interested in using technology to support mental health needs. The variability in type and quality of technology owned by participants suggests the need to design for a range of functionality in the development of mental health tools. Findings also suggest thinking broadly about using existing platforms and widely available tools to support consumers in mental health recovery.

## Introduction

People with mental illnesses in the United States and globally have increasing access to mobile technology [1-4], although some are still unable to avail themselves of these resources [5]. As access grows, so does interest in using technology tools to deliver mental health services; thus, mental health apps are quickly becoming available. These apps are designed for many purposes, from providing information, to self-management, to evidence-based therapies such as cognitive behavioral therapy [6-9]. A few specific tools developed for people with serious mental illnesses (SMIs; ie, schizophrenia, bipolar disorder, and depression) include apps for symptom assessment [10], self-management of psychiatric symptoms [11], remote sensing of behaviors to predict relapse [12], improving medication management through shared decision making [13], and work support [14]. Mental health apps have shown promise in addressing several barriers that exist with traditional mental health services. They offer a platform for increasing access to evidence-based interventions and providing services to underserved populations. [5-8,15,16].

Accumulating evidence on technology use by people with SMI has found that those with SMI use technology in comparable ways to the general population for communication, social connection, and access to information, including health-related information [17-20]. Our research builds on these survey-based findings by applying ethnographic and qualitative methods to elucidate details and nuances of technology use among people with mental illnesses that may be difficult to discern via other modes of inquiry. For example, aggregate numbers tell us that people with mental illness have widespread access to technology, but what is it like for them, and do they experience challenges not yet identified in the literature? What is the range of technologies being used to address mental health needs? How do orientations to, and use of, technologies vary across mental health service delivery settings? With such questions as points of departure, in this qualitative study, we examined current practices and orientations toward technology among service users within 3 mental health settings.

## Methods

The study was conducted in 3 mental health settings in the Northeastern United States. Sites were selected to represent a range of service settings (ie, private and community, outpatient and residential) and people from diverse socioeconomic backgrounds and ages. The study sites included a private dual diagnosis clinic for young adults with early psychosis and co-occurring addiction; a private residential treatment program serving a predominantly older adult population with long-term mental disorders; and an academic-affiliated community mental health center serving a broad population with mental illnesses. The location of the mental health centers included urban and rural settings.

Service users at the private clinic are typically young adults of higher socioeconomic status, who are single and employed or pursuing postsecondary education. Service users at the private residential treatment program are typically middle-to older-aged adults of middle to high socioeconomic status, who are single, unemployed, and long-term recipients of residential or inpatient mental health care. Service users at the community mental health center are typically unemployed, single, and of low socioeconomic status, who vary in age from young to older adults. The two private centers each served 40-50 people at any given time; the community mental health center served approximately 1500 adults with mental illness.

This multimethod study combined ethnographic observation and qualitative interviewing to inquire into use of, and orientations toward, technology. We defined technology broadly to include personal devices such as mobile phones (smartphones and nonsmartphones), computers, and tablets as well as technology made available through mental health organizations such as videoconferencing and public computers. These are examples of technology and not an exhaustive list; we were attuned to a wide range of technology use among participants—from hard-line telephones and cassette players to the latest tablets and mobile phones. The scope of technologies observed was intentionally broad to provide a more complete understanding of the range of technologies currently used by people with mental illnesses.

Data were collected over 1 year, during which we engaged with each site for 3-4 months. The study was approved by our university’s research ethics committee, and participants gave informed consent to participate. Participant observation was the primary method employed in earlier stages of the research in the 2 private treatment settings. Ethnographic methods are particularly well suited for initial explorations into a new area of research. In each of the private centers, most current service users and staff were included in the study, that is, we did not sample at the individual level. Instead, purposive sampling occurred at the *site level* to a range of settings serving people with mental illnesses who could provide insight into technology use. In these settings, the research team built awareness of the study through Information Sessions in which the research team introduced themselves and provided an overview of the study to service users and staff. Participants could “opt out” of participating, meaning that the research team would not interact directly with them or take notes about their behavior or interactions. No person opted out in either setting.

Ethnographic visits were half-day to daylong visits at each site occurring weekly for 3-4 months. During this time, the ethnographic researcher interacted with key stakeholders, including service users, frontline staff, supervisors, and leadership within the organization, and became familiar with the organizational environment and culture. The potential influence of the researcher on behaviors in the organization was diminished by becoming a regular presence in the setting. During ethnographic visits, the researcher was positioned as both a participant and observer, immersing herself in the setting and sharing the daily lives of participants while also remaining attentive to the aims of the research [21,22]. The researcher observed and interacted with service users and staff at multiple venues, including clinical offices, community-based visits, events and activities, and common areas within the clinic and residential settings. This yielded many opportunities to observe if and how various forms of technology were salient. In addition, informal interviews were conducted with service users and staff that provided a basis for open-ended inquiry about use of, and interest in, technology. Detailed fieldnotes were written following ethnographic visits to systematically document behaviors and interactions in the setting, with particular attention to use of technology and dialogue regarding technology.

As the research progressed, we used the exploratory observations and informal interviews from the ethnography to inform and design more focused research interactions. In the community mental health center, three of the authors (ECS, VAN, SCA) conducted brief, semistructured interviews with mental health service users. Interviews were conducted until the team felt confident that similar ideas and provisional patterns were recurrent in the dataset, at which point no additional participants were enrolled. The final sample for the interviews included 15 participants. Interviews were organized around the following domains: use of technology, interest in technology, technology in mental health services, and technology and mental health recovery. Interviews were conducted in a private office at the community mental health center, were 15-20 minutes in duration, and were audiorecorded and transcribed.

We reviewed and coded fieldnotes and interview transcripts. Qualitative coding is a process of tagging portions of text with a meaningful label [23]. Coding is the “pivotal link” between data collection and interpreting the meaning of qualitative data [23]. We developed qualitative codes from both researcher- driven categories derived from the research aims and interview questions as well as categories that arose through inductive review of the qualitative data. Coding was done iteratively and involved multiple researchers. Key concepts and patterns were identified through continued immersion in the dataset. The research team met regularly to discuss provisional findings and also received feedback via expert review. The team worked collaboratively to refine and reach consensus on the main findings reported in this article.

## Results

### Technology Owned

We found variability across mental health service delivery settings by type of technology owned, as well as by awareness of, and interest in, technology; and routine uses of technology. In the private dual diagnosis center that primarily served young adults, participants typically owned several high-end devices, including luxury brand (eg, Apple, Samsung) smartphones, tablets, and computers. In contrast, in the long-term residential care center, a few participants owned modern, high-end devices, but the majority owned outdated technology, for example, old flip phones tucked away in desk drawers and outdated laptops left uncharged or broken. In the community mental health center setting, all participants owned at least one modern device—commonly a smartphone—yet these were typically low-budget, prepaid mobile plans with limited data.

Across all 3 sites, participants’ access to technology was facilitated or constrained by various factors. In some cases, policies at the organization prevented participants from using technology, especially when substance use challenges were present. For other participants, access to technology was mediated by whether family members supported their use of technology. Some family members encouraged using technology, citing reasons ranging from carrying a cell phone for safety to the desire for the participant to develop computer skills. Yet other families had dismissed participants’ desire to own technology stating, “You don’t need it.” Finally, access to technology was facilitated or constrained by whether the participant had adequate financial resources to purchase and maintain technology.

### Awareness of, and Interest in, Technology

In both the private dual diagnosis center and the community mental health center, participants had high awareness of the existence of a wide range of technologies even if they did not own the specific devices. They generally were aware of key features and functions of modern technologies (eg, texting; accessing the internet; daily-use apps such as calendar, email, clock, and weather). Similarly, most participants in these 2 settings expressed interest in learning to use technology generally and to support their mental health.

Technological awareness among participants in the long-term residential care center, by contrast, varied. While a few participants in this setting had high awareness, many others were unaware of existing technologies. For example, several people did not know what an iPad was. Similarly, awareness of technological functions was limited in this setting. One older man used the ethnographer’s phone to engage in texting for the first time. Interest in technology among participants in this setting also varied. Some participants expressed interest in learning about and using technology. For example, the participant who texted for the first time was quite enthusiastic about the experience. He immediately saw the potential of texting in making his regular communication (currently via letters) with family and friends much easier. However, for other participants at the long-term residential care center, interest in technology was low as exemplified by the following statements: “I’ve lived my whole life without it” and “I don’t want this.”

### Routine Uses of Technology

Across all 3 settings, participants who engaged with technology were using devices for a wide range of purposes. Participants in the private dual diagnosis center were active and regular users of a range of technology for education, research, communication, social networking, relaxation, and entertainment. For example, participants used Web-based videos to learn new skills such as playing the guitar; others were engaged in formal Web-based college courses. Several participants in this setting enjoyed streaming movies, playing video games, and Web-based shopping. Participants in this setting were all familiar with social media, but varied in their engagement with these platforms depending on whether social media was a positive or negative experience for them. Some people found positive, inspirational information and connections on social media, while others had negative reactions to references to partying or the influx of news about troubling current events. With respect to using technology for mental health-related reasons, this was only observed in one participant who used the alarm on his cell phone as a reminder to take his medication.

In the long-term residential care center, the few participants who regularly engaged with technology used their devices for education, relaxation, and entertainment. Participants who owned functioning computers used the internet to access news, email, and stream movies. A few participants in this setting used social media to connect with family and friends and a few enjoyed playing video games. Participants were also observed using older forms of technology such as cassette tapes and video home system (VHS) movies for relaxation and entertainment. No participants in the long-term residential center were observed or reported using mental health-related technology, for example, mobile health (mHealth) apps, or used technology for mental health-related reasons such as symptom management.

Participants in the community mental health center all actively used technology for a range of purposes, including education, research, social networking, relaxation, and entertainment. As with participants in the other settings, those at the community mental health center commonly used the internet for email and to access information, including current events, recipes, weather, and health. Some participants searched for jobs via Web-based sources; other participants used social media regularly. Many enjoyed streaming movies and listening to music on their cell phones or laptops. In contrast to the other settings, participants in the community mental health center commonly discussed using technology for health and mental health-related purposes. Accessing health-related websites for information was a common practice, as described by the following participant:

I use [my phone] for internet, looking up prescriptions, I use it for diagnoses…I just google it. And I just type in the type of medicine, or the specific name of the medicine…I look it up for side effects. Basically to see if it coincides with the paper I get through a pharmacy. And like, accidental overdose, any interactions, anything like that. And what exactly the medicine does.

Others reported using technology for psychiatric symptom management and to support recovery. Although some people were aware of or had tried mental health apps, these were not commonly used. Participants described some barriers to using mental health apps, including difficulty understanding some apps:

Well I downloaded a couple. And then. It was like measuring my depression. But see, mine goes up and down. And, I really didn’t understand how to use it.

This participant elaborated that if there were an easy-to-understand app for bipolar disorder, he would “definitely use it.” Another participant described how limited data plans prevented her from exploring available mental health apps:

There have been times I think people have suggested, “Check this app out, check that app out,” and for the most part I don’t think I have. That’s one thing I am limited with the phone. I do only have so much data. So, that does limit me some. Ok, do I really want to waste data on looking this up, or getting this app? Or do I want to be able to listen to music?

Rather than using specific mental health apps, participants in the community mental health center reported using widely available and popular apps in ways that supported their mental health. For example, one woman used Instagram every day to access positive affirmations, expressing that this daily practice supported her mental health recovery:

And it helps. Definitely with my depression. Some with the anxiety. If you really start looking at positive affirmations and really start reading them.

Similarly, another participant described using YouTube videos to manage panic attacks and to help with sleep:

I do use a lot of YouTube [videos]. Like I do progressive muscle relaxation when I’m having panic attacks. I also use it for music. To fall asleep to. I’ve had quite a few apps for anxiety and stuff that haven’t worked.

## Discussion

### Principal Findings

We used an ethnographic and qualitative approach in multiple mental health settings to contribute to the growing knowledge base regarding technology and mental health. Our findings are consistent with previous research that has found that people with SMI use technology in a range of ways similar to the general population [17-20]. Previous research has found lower rates of smartphone usage among people with SMI compared with the general population [19,24]. Similarly, we found that although modern technologies, including mobile phones, were commonly owned by participants, ownership was far from ubiquitous in certain settings. Our research contributes to identifying subpopulations that may be less likely to own modern devices and less familiar with the capabilities of modern technology. In particular, our findings suggest that older populations in long-term care settings may need education and support to increase awareness of technology before introducing technology-based supports for mental health. In addition, our findings underscore that access to technology occurs in a social context, and family members may also need some information to become aware that technology may be beneficial for supporting mental health.

Our research extends previous research on technology use among people with mental illnesses by identifying challenges regarding the range of devices owned. We found that owning a device did not necessarily confer full access to available technology-based resources. Many participants owned outdated devices or devices with limited functionality. The low-budget smartphones commonly owned by participants in the community mental health center had limited data plans and storage, which inhibited participants’ ability to download and use mobile apps. These findings hold implications for the development of technology-based mental health supports that are inclusive and can be broadly implemented. Our findings illuminate that mental health app developers should expect—and design for—a range of technological functionality. Facilitating access to technology-based supports in real-world settings will necessitate developing tools that are ecologically valid and take into account limitations posed by outdated or low-budget technology. Current mHealth research may obscure these challenges due to the common practice of providing luxury phones with large data plans to study participants.

In the settings of this study, technology-based tools specific to mental health were not in routine use. This suggests that despite the vast number of health apps currently available for download [25], at this time, mental health service users may not be routinely seeking out and using such tools. In the community mental health setting, we identified some creative uses of widely available apps to support mental health. Our study suggests thinking broadly about and evaluating a range of apps—including, but not limited to—mental health apps, for example, coloring apps, brainteaser apps, and day planner apps. Prior to recommendation, any app would need to be evaluated because many apps contain information that is inconsistent with current practice guidelines [9] and some contain harmful information [26]. We see potential for applying an evaluation framework [27] to facilitate practical and informed decision making around a range of apps in clinical contexts.

Several strategies have shown promise in addressing barriers to the uptake of technology-based mental health supports. Facilitating the routine use of these supports will likely require a multipronged approach. This approach might include Web-based collections of evidence-based tools to make it easier for people to find high-quality supports [28]; educational or informational interventions to increase the engagement and interest of people regarding technology-based tools [29-31]; and government endorsement, systems of accreditation, and funding to increase the availability of high-quality technology- based tools [31]. Because mental health clinicians and service users are relatively naïve about using technology to support mental health, mental health programs may need to include some form of specific expertise to help the two parties find appropriate, effective tools and learn how to use them, at least over the short run [32].

Qualitative inquiry using multiple methods (ethnographic observations and interviews) at multiple sites over 12 months enhanced the credibility and transferability of our qualitative findings [33]. However, like most qualitative research, our findings are not broadly generalizable. Our study was also limited by a lack of ethnic or racial diversity in our sample.

### Conclusions

Many mental health service users currently use technologies but not often in service of addressing mental health needs. Our findings suggest that many, but not all, service users will be interested in using technology to support their recovery. Technology-based mental health education, supports, and interventions should be among the many services offered within mental health centers. Future research should examine the use of technology to support mental health service users and clinicians in real-world settings and also among populations less well connected to services.

## Abbreviations

mHealth: mobile health
SMI: serious mental illness
